# A Transcultural Perspective of Systemic Lupus Erythematosus–Related Fatigue: Systematic Review and Narrative Synthesis

**DOI:** 10.2196/39132

**Published:** 2022-09-15

**Authors:** Jerick Tabudlo, Leorey Saligan

**Affiliations:** 1 College of Nursing University of the Philippines Manila Manila Philippines; 2 National Institute of Nursing Research National Institutes of Health Bethesda, MD United States

**Keywords:** systemic lupus erythematosus, fatigue, transcultural, cross-cultural, multicultural, ethnic, multi-cultural, perspective, perception, attitude, systematic review, narrative synthesis, review, lupus, autoimmune, chronic disease, chronic condition, joint pain, inflammation

## Abstract

**Background:**

Fatigue is one of the most common symptoms of systemic lupus erythematosus (SLE) worldwide, yet it remains poorly assessed and managed. The lack of universal definition and standard measurement of fatigue may add to the continued limitations in its understanding across cultures.

**Objective:**

The psycho-sociocultural underpinnings of fatigue are understudied; therefore, in this paper, we conducted a systematic review to understand a transcultural perspective of SLE-related fatigue.

**Methods:**

Following PRISMA (Preferred Reporting items for Systematic Reviews and Meta-Analysis) systematic review guidelines, we searched CINAHL Complete, Scopus, and PubMed databases for all published articles covered until the search date. Search was expanded using citation and web search. A 3-step process was used to identify articles meeting the inclusion criteria. The results were analyzed using narrative synthesis.

**Results:**

From a total of 370 (n=364, 98.4% scientific databases; n=6, 1.6% web and citation search) articles searched, 18 (4.9%) studies met the inclusion and exclusion criteria and were included in this review. All (18/18, 100%) studies enrolled primarily female participants, and half (9/18, 50%) had cross-sectional designs. Although race was not reported in all studies, most studies had White racial background as the largest proportion of their samples. A majority (7/18, 39%) of the studies were conducted in the United States. Using a narrative synthesis, the prominent themes drawn based on the domains of the culture care theory (CCT) and the sunrise enabler were as follows: SLE-related fatigue (1) as an integral component of the disease process, (2) as a personal challenge, and (3) as a psychosocial dimension.

**Conclusions:**

CCT and sunrise enabler by Leininger guided this review. There are still gaps on how other domains of the CCT and sunrise enabler might influence SLE-related fatigue experience, assessment, and evaluation. The findings from this review showed that SLE-related fatigue has disease, personal, and psychosocial components. Thus, a purely subjective assessment of fatigue in SLE and even other conditions may limit a more accurate assessment and management. The inclusion of disease, personal, and psychosocial indicators is warranted and essential. A culturally sensitive and congruent assessment as well as evaluation models and measurement tools should be developed to capture fatigue experiences accurately. In addition, since global migration is inevitable, advancement in symptom management strategies should coincide with the understanding that fatigue has subjective and objective indicators present across cultures.

## Introduction

Due to the increase in global migration, health care organizations are caring for more culturally diverse individuals and families. This culturally diverse setup is likewise expanding in the health care workforce, especially in countries recruiting foreign-educated and trained health professionals. These inevitable changes in the current landscape may influence the accurate assessment of symptoms and provision of culturally congruent care, especially in fatigue, which has robust psycho-sociocultural underpinnings but is currently without universal definition or standard of measurement. Given this situation, a better understanding of fatigue across cultures is needed.

Although there is no universal standard measurement of fatigue [1], it can be defined as an overwhelming, uncommon, extreme tiredness [2]. In patients with systemic lupus erythematosus (SLE), fatigue is the most common and most prevalent symptom. Fatigue is present even with mild and inactive cases of SLE disease [3]. Although numerous studies and reviews highlighted SLE-related fatigue as the most burdensome and often the most reported symptom in SLE, it remains poorly assessed and managed across cultures. Since there is no cure for SLE, quality of life may be improved with accurate assessment, evaluation, and management of SLE-related fatigue. SLE-related fatigue is commonly associated with other symptoms that may compound limitations to individuals with SLE. This article aimed to conduct a systematic review to develop a transcultural perspective of SLE-related fatigue.

A transcultural perspective integrates similarities and differences of certain cultures to provide culturally congruent health care [4]. Thus, an integrated transcultural perspective of SLE-related fatigue from empirical studies may facilitate a more accurate and culturally congruent symptom assessment, evaluation, and management. Although fatigue severity can be validly and reliably measured in SLE [5,6], a transcultural perspective provides a better understanding because the social and cultural factors (eg, health beliefs) may also determine the threshold for the symptom’s normality and pathology [7], especially when caring for individuals across cultures. The social and cultural structures identified in the culture care theory (CCT) and the sunrise enabler influence health patterns and well-being [8].

This article presents a transcultural (nursing) perspective. This perspective aims to provide culturally congruent care and practice based on the domains of the CCT and sunrise enabler, such as “technology, religion, family and kinship, politics, cultural beliefs and practices, economics, physical conditions, and biological factors” [8].

## Methods

### Search Strategy

A search strategy was developed in consultation with subject matter experts using Medical Subject Headings (MeSH) terms relevant to SLE-associated fatigue and cultural perspectives of fatigue (Textbox 1). We searched for all published articles meeting inclusion criteria in scientific databases such as CINAHL Complete, Scopus, and PubMed until the search date (October 14, 2021). Table 1 presents the search strategy used. We used the same terms in our web search; however, no search terms were used for the citation-based search. Citation-based search means finding articles based on what a related article has referenced.

**Review question and Medical Subject Headings (MeSH) terms. Primary review question: What is the transcultural or cross-cultural perspective of SLE-related fatigue?**

**MeSH**

Systemic lupus erythematosus, lupus erythematosus disseminates, or Lupus AND Fatigue AND Culture, Transcultural, and “Cross-cultural Comparison”

**Keywords**

“systemic lupus erythematosus” OR “lupus erythematosus disseminatus” OR “libman-sacks disease” OR “libman sacks disease” OR “disease, libman-sacks” OR “lupus” AND fatigue OR exhaust* OR tired* OR lethargy OR “muscle weak*” AND cultur* OR transcultur* OR “cross-cultural comparison”

**Table 1:** Search strategy and search outcome (N=364).

Databases	Search strategy	No. of records, n (%)
CINAHL Complete	“systemic lupus erythematosus” OR “lupus erythematosus disseminatus” OR “libman-sacks disease” OR “libman sacks disease” OR “disease, libman-sacks” OR “lupus” AND fatigue OR exhaust* OR tired* OR lethargy OR “muscle weak*” AND cultur* OR transcultur* OR “cross-cultural comparison” OR belief* OR value* OR attitude* Limiters - Peer Reviewed	59 (16.2)
Scopus	“systemic lupus erythematosus” OR “lupus erythematosus disseminatus” OR “libman-sacks disease” OR “libman sacks disease” OR “disease, libman-sacks” OR “lupus” AND fatigue OR exhaust* OR tired* OR lethargy OR “muscle weak*” AND cultur* OR transcultur* OR “cross-cultural comparison” OR belief* OR value* OR attitude*	174 (47.8)
PubMed	“systemic lupus erythematosus” OR “lupus erythematosus disseminatus” OR “libman-sacks disease” OR “libman sacks disease” OR “disease, libman-sacks” OR “lupus” AND fatigue OR exhaust* OR tired* OR lethargy OR “muscle weak*” AND cultur* OR transcultur* OR “cross-cultural comparison” OR belief* OR value* OR attitude*	131 (36)

### Inclusion and Exclusion Criteria

Articles were included if (1) they were original peer-reviewed research articles, (2) they were published in English, (3) they focused on patients with SLE, and (4) they mentioned fatigue and its derivative terms in the title or abstract. All research designs were included. Articles were excluded (1) if the articles did not describe a cultural perspective based on the CCT and the sunrise enabler or (2) if the articles were commentary, abstracts, theoretical, quality improvement projects, or literature review articles.

### Study Selection

Articles were directly downloaded using the comma-separated values format. The downloaded comma-separated values, which is in Microsoft Excel, assisted the authors with organizing and deduplication. We used a three-step screening process, which is as follows: (1) titles were screened for relevance to the research question; (2) abstracts were then screened to determine further if inclusion criteria were met; and (3) full-text screening was completed on all articles accepted in step 2. Additional articles were also included using web and citation searches. Screenings were conducted independently by JBT and LNS. Disagreements were resolved through discussion. The more senior author (LNS) made the final decision if consensus was not reached. Table 1 presents the search outcome.

### Data Abstraction

A data charting form was developed based on PRISMA (Preferred Reporting items for Systematic Reviews and Meta-Analysis) guidelines by JBT and calibrated by both JBT and LNS. JBT extracted the data. LNS performed a quality check on data extraction after data extraction was complete. Based on the extracted information, homogeneity could not be established since the findings were not similar, and therefore meta-analysis or quantitative pooling was not feasible. We did not specify to which domains the findings belong because we maintain that a transcultural perspective is always open to interpretation from various cultures. Thus, by understanding the domains of CCT and the sunrise enabler by Leininger, a transcultural perspective from the findings were extracted.

### Quality Appraisal

Quality appraisal was conducted using the Joanna Briggs Institute Critical Appraisal Checklists for Analytical Cross-Sectional Studies [9]. The checklist contains 8 items that assess the risk of bias in studies. The 2 authors independently appraised each article. Disagreements were adjudicated by the more senior author (LNS).

### Data Synthesis

We synthesized extracted data using a thematic approach. This type of synthesis is useful when the data are varied and when dealing with abstract findings. Specifically, thematic synthesis involves an iterative review of data to identify patterns or themes. The authors used a narrative approach of thematic type for the following reasons: (1) meta-analysis was not feasible; (2) the main topic dealt with an abstract topic (transcultural perspective); and (3) data extracted from the included studies contained more textual data rather than numerical data.

## Results

### Study Selection

**Figure 1:**
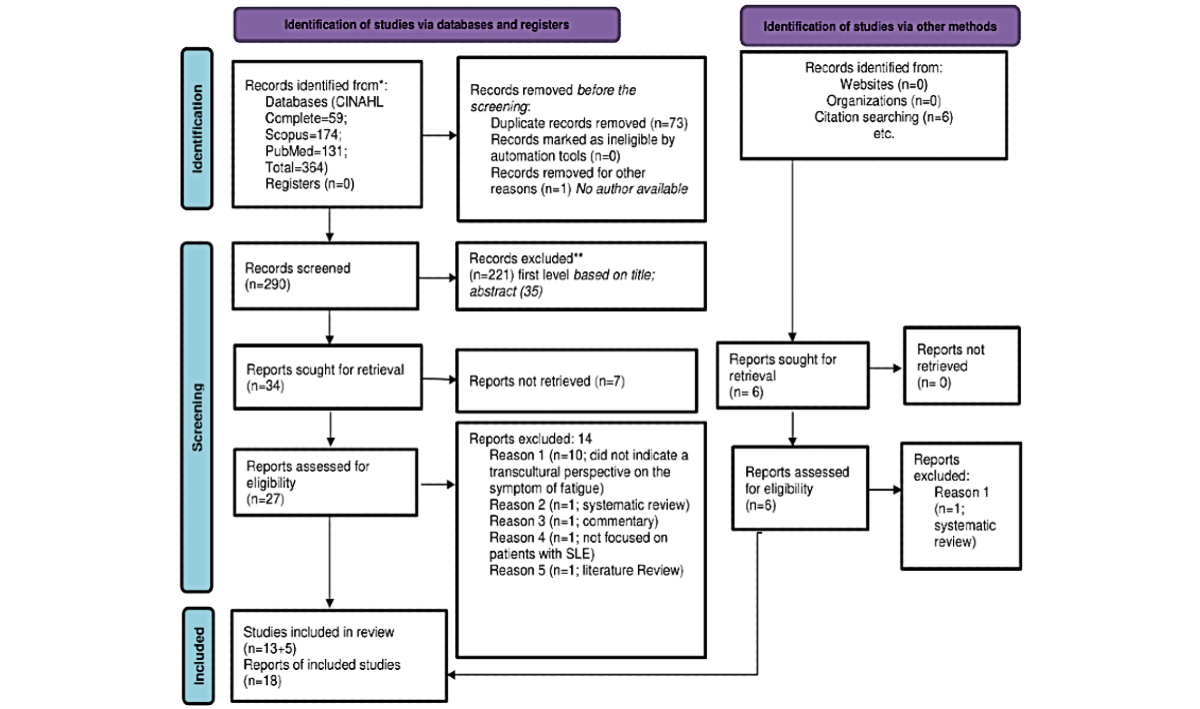
PRISMA (Preferred Reporting Items for Systematic Reviews and Meta-Analyses) 2020 flow diagram, which includes search of databases, registers, and other sources. SLE: systemic lupus erythematosus. *Consider, if feasible to do so, reporting the number of records identified from each database or register searched (rather than the total number across all databases/registers). **If automation tools were used, indicate how many records were excluded by a human and how many were excluded by automation tools.

A total of 364 articles were initially identified. After deduplication of these 364 articles, 290 (79.7%) underwent title and abstract screening; of these, 27 (9.3%) were eligible for full-text screening. In step 3, of the 27 eligible articles, 14 (52%) were excluded (Figure 1). Moreover, we identified 6 additional articles during the citation and web search; of these 6 articles, 1 (17%) was excluded (Figure 1), resulting in a final sample of 18 articles.

### Study Characteristics

The details regarding article characteristics included in this review are in Table 2. Briefly, 9 countries were represented, the United States [10-16], Canada [17,18], Sweden [19,20], Ireland [21,22], the United Kingdom [23], Denmark [24], Puerto Rico [25], Australia [26], and South Africa [27]. The participants in these studies were predominantly female and of White racial backgrounds. Of the 18 studies, 9 (50%) used cross-sectional designs [10-12,15-17,20,23,26], 4 (22%) were qualitative [13,19,24,27], 2 (11%) used mixed-methods [14,21,22], and 2 (11%) were longitudinal studies [18,25].

**Table 2:** Study characteristics and the related findings of the studies included.

Author, year, and country	Title	Design and objectives	Sample	Transcultural fatigue perspective	Other key findings
Almehed et al [20], 2010, Sweden	Health-related quality of life in systemic lupus erythematosus and its association with disease and work disability	Cross-sectional study: to determine the quality of life and its association with disease variables, employment status, and vertebral fracture among women with SLE^a^.	N=163; mean age: 48.5 (SD 13.6) years, sex: 100% female; race or ethnicity: “Predominantly Caucasian”	Patients with SLE scored significantly lower than controls on all SF-36^b^ subscales. SF-36 is a tool to assess HRQoL^c^ and a frequently cited general tool to measure fatigue.	The physical component score of SF-36 is associated with working ability, low SLEDAI-2K^d^, glucocorticosteroid dose, and BMI. Being able to work was also significantly associated with younger age and high scores in PF^e^ and RP^f^.
Burgos et al [25], 2009, United States and Puerto Rico	Disease Activity and Damage Are Not Associated With Increased Levels of Fatigue in Systemic Lupus Erythematosus Patients From a Multiethnic Cohort: LXVII	Longitudinal multiethnic cohort study: to determine the factors associated with increased levels of fatigue symptom through the disease course among patients with SLE.	N=515; mean age: 37.2 (SD 12.6) years; sex: 90.5% female; race or ethnicity: 32.8% African Americans	Increased fatigue levels were associated with White ethnicity; constitutional symptoms such as fever and chills, higher levels of the pain experience, abnormal illness-related behaviors, and helplessness. At the same time, the exercise showed to be associated with lower fatigue levels. Using SF-36, lower physical component score and mental component score, higher levels of helplessness, and abnormal illness-related behaviors were associated with higher fatigue levels. While higher levels of social support and lower levels of fatigue show association.	Demographics and socioeconomic factors were not significantly associated with fatigue. In addition, clinical characteristics were not associated with the (higher or lower) fatigue levels.
Connolly et al [21], 2014, Ireland	Fatigue in systemic lupus erythematosus: impact on occupational participation and reported management strategies	Exploratory study (descriptive statistics and qualitative descriptive guidelines): to explore how people with SLE describe and cope with fatigue in their everyday lives.	N=12; mean age: 14.3 (SD 10) years; sex: 91.67% (11/12) female; race or ethnicity:100% White	In the study, fatigue was described as an unpredictable and constant characteristic of SLE, which affects work, leisure, and occupation. The most common factors to increase fatigue among the participants were stress, physical activity, and joint pain. Stress is the most commonly expressed concern, along with the experience of fatigue.	Social support from family, friends, and neighbors was valuable and provided practical help. Participation in employment requires routine and environmental modifications and considerable flexibility.
Da Costa et al [17], 2006, Canada	Dimensions of Fatigue in Systemic Lupus Erythematosus: Relationship to Disease Status and Behavioral and Psychosocial Factors	Cross-sectional study: using a multidimensional assessment, the study aimed to characterize the experience of fatigue in patients with SLE. It also determined the contributors to the physical and mental aspects of fatigue.	N=130; mean age: 45.4 (SD 14) years; sex: 100% female; race or ethnicity: “Primarily Caucasian”	In the study, there were significant positive correlations between physical fatigue, disease activity and damage, the presence of fibromyalgia, depression, and impaired sleep quality. There were also significant negative correlations between social support satisfaction and exercise.	Greater disease damage and disease activity; the presence of fibromyalgia, depressed mood, sleep disturbance, and less participation in leisure-time physical activity contributed to higher physical fatigue scores.
Dobkin et al [18], 2001, Canada	Living with Lupus: A Prospective Pan-Canadian Study	Prospective study: to portray the life of women with lupus and identify the predictors to the symptom of fatigue.	N=120; mean age: 42.50 (SD 10.83) years; sex: 100% female; race or ethnicity: 84.2% White	Patients experienced less fatigue with decreased depression and stress.	In the final follow-up of the study, even though the majority is faring in terms of distress, there remains a subset who experience distress and may benefit from psychosocial interventions.
Donnelly et al [10], 2018, United States	Fatigue and Depression Predict Reduced Health-Related Quality of Life in Childhood-Onset Lupus	Cross-sectional study: to identify the risk factors that persistently reduced patients’ health-related QoL^g^ with childhood-onset lupus. It also described a risk profile for persistent reduced health-related QoL.	N=50; mean age: 16.2 (SD 2.5) years; sex: 84% female; race or ethnicity: 23 (46%) African American; 23 (46%) White	At follow-up, poorer HRQoL was significantly predicted by higher fatigue symptoms and depressive symptoms during the initial visit.	A profile of significant anxiety, greater pain, and coping difficulties was seen in the high-risk group.
Jump et al [11], 2005, United States	Fatigue in Systemic Lupus Erythematosus: Contributions of Disease Activity, Pain, Depression, and Perceived Social Support	Cross-sectional study: to investigate the associations between pain, depression, fatigue, and disease activity in patients with SLE.	N=127; mean age: 40.6 (SD 12.2) years; sex: 100% female; race or ethnicity: 62 (48.8%) White	There was an inverse relationship between perceived social support and fatigue. Perceived social support accounted for an additional 4% of the variance in fatigue scores.	Fatigue was the most rated symptom, significantly higher than all other symptoms assessed.
Kent et al [23], 2017, United Kingdom	Burden of illness in systemic lupus erythematosus: results from a UK patient and carer online survey	Cross-sectional web-based survey: to assess the impact of SLE on both patients and carers.	N=121; age: 34% from 41-50 years median age group; sex: 94% female; race or ethnicity: 77% White	The symptom of fatigue and the feeling of being a burden to others greatly influenced the patient's HRQoL. In addition, the patient's social interactions are affected. Patients also reported reduced ability to engage in social activities (N=121, 89%).	Fatigue was the most debilitating symptom experienced daily by 79% (N=121) of patients. Patients with SLE relied heavily for help on their carer with basic daily chores, and 62% (N=121) also required emotional or social support.
Kier et al [24], 2016, Denmark	How do women with lupus manage fatigue? A focus group study	Explorative qualitative study (focus group study): to describe the way patients with SLE manage their experience of fatigue.	N=27; median age: 53 (range 26-72) years; sex: 100% female; race or ethnicity: White	The main themes covered were related to being open, listening to the body, and accepting the experience of fatigue.	Fatigue is considered a controlling factor in everyday life of women with SLE.
Kozora et al [12], 2005, United States	Major life stress, coping styles, and social support in relation to psychological distress in patients with systemic lupus erythematosus	Cross-sectional study: to examine the psychological processes in patients with SLE and rheumatoid arthritis with the measures of life stress, coping styles, social support, and cognitive ability.	N=52 patients with SLE; mean age: 34.6 (range 18-57) years; sex: 90.38% (n=47) female; race or ethnicity: 56.9% White in SLE group; 86%-96% White in the RA^h^ and control groups	Patients with SLE had greater distress (which included fatigue) than patients with rheumatoid arthritis and healthy controls on all the subscales and the total score.	Patients with SLE had a significantly higher Social Withdrawal Subscale score from the Coping Style Inventory compared with healthy controls.
Mattsson et al [19], 2012, Sweden	Uncertainty and Opportunities in Patients with Established Systemic Lupus Erythematosus: A Qualitative Study	Qualitative study: to describe the experience in everyday life of patients with established SLE; this included the negative and positive aspects.	N=19; median age: 55 (range 27-80) years; sex: 84.21% (n=16) female; race or ethnicity: not reported	Pain and fatigue influenced the everyday life of patients with SLE.	The experience of patients with SLE reflects uncertainties and opportunities.
Moses et al [26], 2005, Australia	Prevalence and correlates of perceived unmet needs of people with systemic lupus erythematosus	Cross-sectional descriptive study: to assess the prevalence and associations of perceived unmet needs of people with SLE.	N=386; mean age: 52.5 (SD 14.4) years; sex: 94% (n=363) female; race or ethnicity: not reported	Results showed that the need for help with tiredness had the highest prevalence (81%).	Reported proportions of unmet daily living issues varied from 17% to 61% for everyday living issues and reading difficulties, respectively.
O’Riordan et al [22], 2017, Ireland	Fatigue and Activity Management Education for Individuals with Systemic Lupus Erythematosus	Sequential explanatory mixed methods design: to assess the impact of an occupational participation and fatigue management program called FAME.	N=21; mean age: 48.1 (SD 15.25) years; sex:100% female; race or ethnicity: not reported	Participants expressed a lack of understanding of their experience of fatigue. However, attendance to the fatigue management program validated through others in the group helped them accept their experience of fatigue.	The program provided a statistically significant improvement in depression and categories of “burden to others” and “fatigue” of the LupusQoL.
Phuti et al [27], 2019, South Africa	Living with systemic lupus erythematosus in South Africa: a bitter pill to swallow	Phenomenology: to explore the lived experiences, perceptions, and unmet needs of South African patients with SLE.	N=25; mean age: 30.9 (range 22-45) years; sex: 100% female; race or ethnicity: 72% (n=18) Black African	Most of the participants talk about their challenges living with fatigue. Fatigue is commonly misunderstood, which negatively affects activities of daily living, work, and sexual well-being.	Similarly, pain is considered a common complaint affecting ADLs^i^, family, and social life.
Raymond et al [13], 2021, United States	Patient Experience With Fatigue and Qualitative Interview-Based Evidence of Content Validation of The FACIT-Fatigue in Systemic Lupus Erythematosus	Qualitative study: to assess the content validity of the FACIT^j^-Fatigue for patients with SLE and explore the experience of patients with SLE-related fatigue.	N=15; mean age: 52.1 (SD 13.1) years; sex: 86.7% (n=13) female; race or ethnicity: 53.3% non-Hispanic or White	Fatigue impacted the participants’ physical functioning (9/15, 60%), emotional impacts (15/15, 100%), social impacts (14/15, 93%), work or school-related roles (12/15, 80%), limited ADLs (15/15, 100%), and unable to do much (14/15, 93%).	The physical difficulties experienced affected their work, while the physical inactivity interfered with their ability to maintain a healthy weight. In addition, being unable to participate in social events was the most frequently reported social functioning limitation.
Robinson et al [14], 2010, United States	Impact of Systemic Lupus Erythematosus on Health, Family, and Work: The Patient Perspective	Phenomenological, mixed-methods approach: to determine the critical health issue of patients with SLE from their perspective.	N=23; mean age: 43 (SD 13) years; sex: 83% (n=19) female; race or ethnicity: 61% (n=14) White	Focus group findings identified the most frequent health issues such as pain (n=19, 83%), fatigue (n=14, 61%), work or school impairment (n=13, 57%). From questionnaire findings, inability to do previous activities (87%), fatigue (87%), pain (87%), and inability to attend work or school (83%).	Arising from the literature review, some of the health issues identified were fatigue, energy, or vitality (n=8); depression (n=7); pain (n=4); helplessness (n=4); and the inability to cope with the disease (n=4). Impaired concentration (n=3), impaired work-life (n=3), anxiety or distress (n=3), and impaired or compromised personal relationships (n=2).
Sterling et al [15], 2014, United States	Patient-reported fatigue and its impact on patients with systemic lupus erythematosus	Cross-sectional qualitative study: to explore the experiences of fatigue among patients with SLE and its impact on their lives.	N=22; mean age: 45.5 (SD 12.52) years; sex: 95% (n=21) female; race or ethnicity: 59% (n=13) African American or Black	Patients reported having variability in nature (frequency and severity). The symptom of fatigue was described to impact emotional, cognitive aspects, ADLs, leisure, as well as social and family activities.	Some participants associated depression with fatigue.
Utset et al [16], 2008, United States	Correlates of Formal Work Disability in an Urban University Systemic Lupus Erythematosus Practice	Cross-sectional study: to determine the demographic, disease-specific, and psychological features associated with work disability among patients with SLE in a medical center.	N=143; mean age: 40.4 (SD 11.6) years; sex: 92% (n=132) female; race or ethnicity: 60.8% African Americans	Fatigue severity scores were significantly worse in formal work disability subjects compared with never-disabled subjects.	Ethnicity was associated with work disability status.

^a^SLE: systemic lupus erythematosus.

^b^SF-36: 36-Item Short Form Survey.

^c^HRQoL: health-related quality of life.

^d^SLEDAI-2K: Systemic Lupus Erythematosus Disease Activity Index 2000.

^e^PF: physical functioning.

^f^RP: role physical.

^g^QoL: quality of life.

^h^RA: rheumatoid arthritis.

^i^ADLs: activities of daily living.

^j^FACIT: Functional Assessment of Chronic Illness Therapy.

### Risk of Bias

In the quality appraisal of the studies included, there are apparent differences in the methodological scores of studies. The scores ranged between 2 and 8 points, which is justifiable since the methodologies are incomparable. Thus, we did not set a cut-off score for inclusion in the review. Based on our appraisal, all studies included information on the criteria for sample inclusion. There was an incomplete discussion of the confounding variables and strategies for dealing with them. In the quality appraisal, the authors rated studies that employed qualitative methodology with “Not Applicable (N/A),” which was reasonable because some of the items in the quality appraisal checklist were not applicable for studies that employed a qualitative methodology.

### Themes

This section presents the narrative synthesis of themes drawn from the studies included. The themes were extracted based on the patterns from the studies following the domains of the culture care theory.

#### SLE-Related Fatigue as Integral to the Disease Process

Based on the studies included in this review, SLE-related fatigue showed variation in the nature and severity in the course of the disease [15]. Greater disease damage and disease activity contributed to higher physical fatigue scores [17]. SLE-related fatigue is also considered an unstable yet constant characteristic of SLE. Its impact ranges from work, leisure, occupation [21], emotional and cognitive aspects, activities of daily living, as well as social and family activities [15]. The common factors to increase fatigue were stress, physical activity, joint pain [21], higher levels of helplessness, and abnormal illness-related behaviors, although exercise is associated with lower levels of fatigue [25]. Thus, SLE-related fatigue can be considered integral to the disease process because it is a common and distinct characteristic of the disease affecting daily functioning and activities. The factors influencing SLE-related fatigue as provided in the literature above reflect the biological, kinship, social, and cultural values, as well as belief and lifeway factors in the domains of the CCT and sunrise enabler.

#### SLE-Related Fatigue as a Personal Challenge

In this review, SLE-related fatigue has been shown to be contributing to the daily challenges of patients with SLE. SLE-related fatigue was considered a controlling factor in the everyday life of women with SLE [24]. It has also been associated with poor Quality of Life (QoL). Patients with SLE had lower 36-Item Short Form Survey (SF-36) scores on all subscales compared with controls [20]. SF-36 is a tool to assess health-related quality of life and is a frequently cited general tool to measure fatigue [28]. In a cross-sectional study, poorer health-related QoL was significantly predicted by higher depressive and fatigue symptoms during the initial visit [10]. SLE-related fatigue, along with pain, influenced the everyday life of patients with SLE [19].

Moreover, SLE-related fatigue is commonly misunderstood, which negatively affects activities of daily living, work, and sexual well-being [27]. Fatigue severity scores were also significantly worse in formal work disability subjects compared with never-disabled subjects [16]. In addition, fatigue and the feeling of being a burden to others had the greatest influence on patients’ health-related QoL [23]. Overall, SLE-related fatigue can be considered a personal challenge because it is associated with a reduced health-related quality of life and daily functioning and has contributed to the daily challenges of individuals with SLE. QoL, as the commonly identified findings associated with SLE-related fatigue, may be related to all the domains of the CCT and sunrise enabler since QoL is a holistic aspect that may influence the domains of the CCT and the sunrise enabler.

#### SLE-Related Fatigue as a Psychosocial Dimension

The psychosocial dimension has influenced SLE-related fatigue’s nature and severity levels. Higher levels of social support were associated with lower fatigue levels [25]. Social support from family, friends, and neighbors of individuals with SLE was considered valuable help while managing their fatigue [21]. There was an inverse relationship between perceived social support and fatigue [11]. Attendance to the fatigue management program of individuals with SLE validated their fatigue experience through others in the group. The program provided a statistically significant improvement in the depression and categories of “burden to others” and “fatigue” using the LupusQoL [22]. Fatigue impacted the participants’ physical functioning (9/15, 60%); it had emotional (15/15, 100%) and social impacts (14/15, 93%); it had also influenced their work or school-related roles (12/15, 80%) and limited activities of daily living (15/15, 100%), and most of the participants (14/15, 93%) reported that they were unable to perform many activities. In addition, being unable to participate in social events was the most frequently reported social functioning limitation [13]. Focus group findings identified the most frequent health issues such as pain (83%), fatigue (61%), and work or school impairment (48%). On questionnaire responses, the findings were similar. The most common health issue was the inability to perform previous activities (87%), pain (87%), and fatigue (87%) [14].

Stress as a social construct also influenced fatigue levels. Patients were less fatigued when depression and stress were decreased [18]. Some participants associated depression with fatigue [15]. Patients with SLE reported fatigue as the most debilitating symptom, which was being experienced daily (N=121, 79%). They also reported reliance on help from their caregivers and a reduced ability to engage in social activities (N=121, 89%) [23]. Patients with SLE experienced more distress compared with patients with rheumatoid arthritis and healthy controls on all the subscales and in total scores. Patients with SLE had significantly higher Social Withdrawal Subscale scores compared with healthy controls [12]. Results showed that the need for help in relation to tiredness had the highest prevalence (81%) [26]. Strong social support is a significant driver in reducing the severity of SLE-related fatigue, while, on the contrary, stress worsens SLE-related fatigue. The CCT domains and sunrise enabler, which represent the findings in this theme, may be related to kinship, social factors, and economic factors.

## Discussion

### Principal Findings

Although SLE-related fatigue is one of the most common symptoms in SLE, there is still an inadequate understanding of it across cultures, which limits providing an accurate assessment, evaluation, and management. Currently, there are no consistent findings on the relationship of SLE-related fatigue and immunologic and inflammatory disease characteristics; however, some psychosocial characteristics are associated with SLE-related fatigue [29]. Following the domains of CCT and the sunrise enabler, this review examined the transcultural perspective of SLE-related fatigue from published empirical studies in identified scientific electronic databases.

The development of the CCT and the sunrise enabler was traced back from Leininger’s dissertation in 1970s, which, as she explained, was derived from anthropology. The theory aims to facilitate and explain the interdependence of culture and care by noting differences and similarities across cultures to achieve a culturally congruent care. Along with the theory is the sunrise enabler, which serves to introduce the different aspects of the theory such as the dimensions, facets, and its components [8]. In the theory, cultural and social structures that influence care expression, patterns and practices, as well as holistic health and well-being are as follows: (1) technological factors; (2) religious, spiritual, and philosophical factors; (3) kinship and social factors; (4) cultural beliefs and lifeways; (5) biological factors; (6) political and legal factor; (6) economic factor; and (7) educational factors [8].

The key findings from this review affirm that SLE-related fatigue has underpinnings rooted in (1) biological factors; (2) kinship and social factors; (3) cultural values, beliefs, and lifeways factors; (4) economic factors; and (5) all factors of the CCT and the sunrise enabler that relate to QoL. Based on the themes of this review, SLE-related fatigue reflects an expanding dimension from a biological point of view to environmental and cultural dimensions.

Often, fatigue is understood from biological factors or from a clinical lens; in this review, the clinical attributes of individual with SLE-related fatigue may represent a biological factor. In the CCT and sunrise enabler, biological factors include hereditary, genetic conditions, including those influencing and being influenced by professional and generic care [8].

Fatigue may be associated with disease activity; however, this is not always the case [30]. In a large international observational study with a systematic review of literature, disease activity showed a weak association with fatigue [31]. Hierarchical multiple regression showed that greater disease damage, disease activity, and other factors contributed to higher physical fatigue scores [17]. Although there are a good number of studies separating fatigue and disease activity, a closer assessment and management of fatigue in all cultures or ethnicities are warranted. In fact, in recent updates of the CCT and sunrise enabler, the biological factors covering culture-bound syndromes as well as the role of genetics and heredity were recognized to affect social and cultural factors [8], which may influence how SLE-related fatigue is expressed and managed. The inclusion of biological factors in understanding fatigue supports the idea that even when mood disorders are absent (common associations of fatigue), fatigue still exists in autoimmune and inflammatory diseases, including SLE [32].

The culture, value, belief, and lifeway factors influencing SLE-related fatigue were the QoL and individual functioning, whereas in terms of kinship and social factors, stress and social support should be taken into consideration. QoL, functioning, social support, and stress are intertwined in a social and cultural setting because they exist in the day-to-day living conditions of individuals with SLE. These findings support those of a prior study that there were significant associations among changes in fatigue scores, SF-36 physical subscale, mood, and some domains of LupusQOL [33], which included pain and burden to others [34]. All LupusQoL domains had low scores with fatigue and being a burden to others as the most affected [35].

A seminal Integrated Fatigue Management Model supports the findings of this review. The dimensions of fatigue, such as subjective, physiological, biochemical and metabolic, and behavioral aspects, are surrounded by social factors such as cultural and ethnic practices, significant life event patterns, environmental patterns, psychological, activity and rest, race, and genetic makeup, among others. The surrounding factors are thought to modulate fatigue [2]. The theory of symptom management also supported the multidimensional aspect of symptoms such as fatigue. The interdependent circles of symptom experience, management strategies, and symptom status outcome interact with the 3 domains of nursing, which include the person domain (demographic, psychological, physiological, sociological, and developmental), environment domain (physical, social, and cultural), and health and illness domain (risk factors, health status and disease, and injury) [36]. Finally, in a concept analysis of fatigue, some of the identified critical attributes of fatigue encompassed physical, cognitive, and emotional dimensions; accordingly, fatigue causes distress and chronic or unrelenting symptoms, and it is dependent on the individual’s perception [37]. Recent developments in symptom clusters also pointed out that symptoms occurring in clusters have strong psycho-sociocultural underpinnings. The psycho-sociocultural factors may mediate or modulate fatigue symptom severity or intensity. Fatigue commonly co-occurs with other symptoms such as in SLE. Although fatigue remains challenging to conceptualize across cultures, this review highlighted the expanding multidimensionality of SLE-related fatigue.

### Limitations

Some of the limitations of this review should be considered. First, a meta-analysis was not feasible due to the variations in methodologies used. With this variation, a narrative summary was deemed more appropriate, and only 3 databases were considered and expanded to citation and web searches. Since only 3 databases were considered, articles from other countries may have been missed. Third, only 1 author (JBT) conducted the synthesis, and it was ascertained by the second author (LNS), which may pose limitations to the breadth of the review. The review may also be limited to the countries where the studies were conducted. For instance, there was no study conducted in an Asian country in this review. An additional limitation of the review may also be pointed to the predominantly female sample and White ethnicity of the study participants enrolled. The name of the research designs was based on what the authors have indicated in their respective articles. Since this review used a thematic approach in presenting the data, this may limit the presentation in terms of the heterogeneity of the studies included. The conclusion may also be biased since the themes were based on a single theoretical framework. There was also an incomplete discussion of the confounding variables in the studies included. Future research or review should also include more male individuals and participants from other racial and ethnic backgrounds and use other theoretical frameworks to comprehensively assess the transcultural aspect of SLE-related fatigue.

### Implications and Conclusions

This systematic review, focusing on SLE-related fatigue, draws evidence that SLE-related fatigue experience has both disease-related, personal, and psychosocial components. Applying culture care theory and sunrise enabler by Leininger, there are still gaps in the literature on how technological advances, individuals’ religion and philosophy, political climate, economic and educational factors, and the worldviews of particular geographic locations might influence SLE-related fatigue experience across cultures. More culturally congruent fatigue symptom assessment and treatment may also be derived by applying the findings of this review. The CCT and the sunrise enabler may be used in different conditions to capture the holistic components of different aspects of the disease. Cultural and contextual considerations are also warranted to improve the assessment and evaluation of the outcomes of SLE-related fatigue. Pure symptom assessment based on subjective data may narrow the potential or actual factors contributing to SLE-related fatigue. Adding the disease, personal and psychosocial indicators of fatigue should be considered. Future research should be conducted on the following areas: (1) large-scale cross-cultural study focusing on contributing factors for SLE-related fatigue; (2) employment impact of SLE-related fatigue; (3) increasing male participants in empirical studies conducted; and (4) transcultural perspective of fatigue in other conditions.

## Abbreviations

CCT: culture care theory
MeSH: Medical Subject Headings
PRISMA: Preferred Reporting items for Systematic Reviews and Meta-Analysis
QoL: Quality of Life
SF-36: 36-Item Short Form Survey
SLE: systemic lupus erythematosus

## References

[1] Kluger BM, Krupp LB, Enoka RM (2013). Fatigue and fatigability in neurologic illnesses: proposal for a unified taxonomy. Neurology.

[2] Piper BF, Carrieri-Kohlman V, Lindsey AM, West CM (1993). Fatigue. Pathophysiological Phenomena in Nursing: Human Responses to Illness.

[3] Elefante E, Tani C, Stagnaro C, Ferro F, Parma A, Carli L, Signorini V, Zucchi D, Peta U, Santoni A, Raffaelli L, Mosca M (2020). Impact of fatigue on health-related quality of life and illness perception in a monocentric cohort of patients with systemic lupus erythematosus. RMD Open.

[4] Leininger M (2002). Transcultural Nursing: Concepts, Theories, Research, and Practices, Third Edition.

[5] Krupp LB, LaRocca NG, Muir-Nash J, Steinberg AD (1989). The fatigue severity scale. Application to patients with multiple sclerosis and systemic lupus erythematosus. Arch Neurol.

[6] Schwartz JE, Jandorf L, Krupp LB (1993). The measurement of fatigue: A new instrument. Journal of Psychosomatic Research.

[7] McLlvenny S (2009). Fatigue as a transcultural issue. European Journal of General Practice.

[8] McFarland MR, Wehbe-Alamah HB (2019). Leininger's Theory of Culture Care Diversity and Universality: An Overview With a Historical Retrospective and a View Toward the Future. J Transcult Nurs.

[9] Moola S, Munn Z, Sears K, Sfetcu R, Currie M, Lisy K, Tufanaru C, Qureshi R, Mattis P, Mu P (2015). Conducting systematic reviews of association (etiology): The Joanna Briggs Institute's approach. Int J Evid Based Healthc.

[10] Donnelly C, Cunningham N, Jones JT, Ji L, Brunner HI, Kashikar-Zuck S (2017). Fatigue and depression predict reduced health-related quality of life in childhood-onset lupus. Lupus.

[11] Jump RL, Robinson ME, Armstrong AE, Barnes EV, Kilbourn KM, Richards HB (2005). Fatigue in systemic lupus erythematosus: contributions of disease activity, pain, depression, and perceived social support. J Rheumatol.

[12] Kozora E, Ellison MC, Waxmonsky JA, Wamboldt FS, Patterson TL (2005). Major life stress, coping styles, and social support in relation to psychological distress in patients with systemic lupus erythematosus. Lupus.

[13] Raymond K, Park J, Joshi AV, White MK (2021). Patient Experience With Fatigue and Qualitative Interview-Based Evidence of Content Validation of The FACIT-Fatigue in Systemic Lupus Erythematosus. Rheumatol Ther.

[14] Robinson D, Aguilar D, Schoenwetter M, Dubois R, Russak S, Ramsey-Goldman R, Navarra S, Hsu B, Revicki D, Cella D, Rapaport MH, Renahan K, Ress R, Wallace D, Weisman M (2010). Impact of systemic lupus erythematosus on health, family, and work: the patient perspective. Arthritis Care Res (Hoboken).

[15] Sterling K, Gallop K, Swinburn P, Flood E, French A, Al Sawah S, Iikuni N, Naegeli A, Nixon A (2014). Patient-reported fatigue and its impact on patients with systemic lupus erythematosus. Lupus.

[16] Utset TO, Chohan S, Booth SA, Laughlin JC, Kocherginsky M, Schmitz A (2008). Correlates of formal work disability in an urban university systemic lupus erythematosus practice. J Rheumatol.

[17] Da Costa Dq, Dritsa M, Bernatsky S, Pineau C, Ménard HA, Dasgupta K, Keschani A, Rippen N, Clarke AE (2006). Dimensions of fatigue in systemic lupus erythematosus: relationship to disease status and behavioral and psychosocial factors. J Rheumatol.

[18] Dobkin PL, Da Costa D, Fortin PR, Edworthy S, Barr S, Esdaile JM, Senécal JL, Goulet JR, Choquette D, Rich E, Beaulieu A, Cividino A, Ensworth S, Smith D, Zummer M, Gladman D, Clarke AE (2001). Living with lupus: a prospective pan-Canadian study. J Rheumatol.

[19] Mattsson M, Möller B, Stamm T, Gard G, Boström C (2012). Uncertainty and opportunities in patients with established systemic lupus erythematosus: a qualitative study. Musculoskeletal Care.

[20] Almehed K, Carlsten H, Forsblad-d'Elia H (2010). Health-related quality of life in systemic lupus erythematosus and its association with disease and work disability. Scand J Rheumatol.

[21] Connolly D, McNally A, Moran D, Ryan M (2014). Fatigue in Systemic Lupus Erythematosus: Impact on Occupational Participation and Reported Management Strategies. British Journal of Occupational Therapy.

[22] O'Riordan R, Doran M, Connolly D (2017). Fatigue and Activity Management Education for Individuals with Systemic Lupus Erythematosus. Occup Ther Int.

[23] Kent T, Davidson A, Newman D, Buck G, D'Cruz D (2017). Burden of illness in systemic lupus erythematosus: results from a UK patient and carer online survey. Lupus.

[24] Kier A, Midtgaard J, Hougaard KS, Berggreen A, Bukh G, Hansen RB, Dreyer L (2016). How do women with lupus manage fatigue? A focus group study. Clin Rheumatol.

[25] Burgos PI, Alarcón GS, McGwin G, Crews KQ, Reveille JD, Vilá LM (2009). Disease activity and damage are not associated with increased levels of fatigue in systemic lupus erythematosus patients from a multiethnic cohort: LXVII. Arthritis Rheum.

[26] Moses N, Wiggers J, Nicholas C, Cockburn J (2005). Prevalence and correlates of perceived unmet needs of people with systemic lupus erythematosus. Patient Educ Couns.

[27] Phuti A, Schneider M, Makan K, Tikly M, Hodkinson B (2019). Living with systemic lupus erythematosus in South Africa: a bitter pill to swallow. Health Qual Life Outcomes.

[28] Ware JE, Kosinski MA, Keller S (1994). SF-36 Physical and Mental Health Summary Scales: A User's Manual. ResearchGate.

[29] Omdal R, Waterloo K, Koldingsnes W, Husby G, Mellgren SI (2003). Fatigue in patients with systemic lupus erythematosus: the psychosocial aspects. J Rheumatol.

[30] Dey M, Parodis I, Nikiphorou E (2021). Fatigue in Systemic Lupus Erythematosus and Rheumatoid Arthritis: A Comparison of Mechanisms, Measures and Management. J Clin Med.

[31] Arnaud L, Gavand PE, Voll R, Schwarting A, Maurier F, Blaison G, Magy-Bertrand N, Pennaforte J-L, Peter H-H, Kieffer P, Bonnotte B, Poindron V, Fiehn C, Lorenz H, Amoura Z, Sibilia J, Martin T (2019). Predictors of fatigue and severe fatigue in a large international cohort of patients with systemic lupus erythematosus and a systematic review of the literature. Rheumatology (Oxford).

[32] Norheim KB, Jonsson G, Omdal R (2011). Biological mechanisms of chronic fatigue. Rheumatology (Oxford).

[33] McElhone K, Abbott J, Shelmerdine J, Bruce IN, Ahmad Y, Gordon C, Peers K, Isenberg D, Ferenkeh-Koroma A, Griffiths B, Akil M, Maddison P, Teh L (2007). Development and validation of a disease-specific health-related quality of life measure, the LupusQol, for adults with systemic lupus erythematosus. Arthritis Rheum.

[34] Bland A, Hunziker S, Barraclough M, Kane K, Cervera R, Dörner T, Doria A, Pouchot J, Stoll T, Penner I-K, Bruce IN (2018). 136: Motor and cognitive fatigue in SLE is associated with mood and health-related quality of life (HRQoL) in patients with SLE: results from the Patient Reported Outcomes in Lupus (PRO-Lupus) study. Rheumatology.

[35] Gordon C, Isenberg D, Lerstrøm K, Norton Y, Nikaï E, Pushparajah DS, Schneider M (2013). The substantial burden of systemic lupus erythematosus on the productivity and careers of patients: a European patient-driven online survey. Rheumatology (Oxford).

[36] Humphreys J, Janson S, Donesky D, Dracup K, Lee KA, Puntillo K, Faucett JA, Aouizerat B, Miaskowski C, Baggott C, Carrieri-Kohlman V, Barger M, Franck L, Kennedy C (2014). 7: Theory of Symptom Management. Middle Range Theory for Nursing.

[37] Ream E, Richardson A (1996). Fatigue: a concept analysis. Int J Nurs Stud.

